# Ultra-High-Performance Liquid Chromatography–Electrospray Ionization–High-Resolution Mass Spectrometry for Distinguishing the Origin of Ellagic Acid Extracts: Pomegranate Peels or Gallnuts

**DOI:** 10.3390/molecules29030666

**Published:** 2024-01-31

**Authors:** Jinchao Wei, Renjian Xu, Yuanyuan Zhang, Lingyu Zhao, Shumu Li, Zhenwen Zhao

**Affiliations:** 1 Beijing National Laboratory for Molecular Sciences, CAS Research/Education Center for Excellence in Molecular Sciences, Key Laboratory of Analytical Chemistry for Living Biosystems, Beijing Mass Spectrum Center, Institute of Chemistry, Chinese Academy of Sciences, Beijing 100190, China; weijinchao@iccas.ac.cn (J.W.); zhangyuanyuan@iccas.ac.cn (Y.Z.); zhaolingyu@iccas.ac.cn (L.Z.); lishumu@iccas.ac.cn (S.L.); 2 Graduate School, University of Chinese Academy of Sciences, Beijing 100049, China; 3 Anhui Deren Biotechnology Co., Ltd., Suzhou 234122, China; xrj.hbzy@163.com

**Keywords:** pomegranate peels, gallnuts, UHPLC-ESI-MS, ginkgolic acid (C15:1), anacardic acid

## Abstract

Ellagic acid, known for its various biological activities, is widely used. Ellagic acid from pomegranate peels is safe for consumption, while that from gallnuts is only suitable for external use. However, there is currently no effective method to confirm the source of ellagic acid. Therefore, this study establishes an analysis method using ultra-high-performance liquid chromatography–electrospray ionization–high-resolution mass spectrometry (UHPLC-ESI-HR-MS) to identify the components of crude ellagic acid extracts from pomegranate peels and gallnuts. The analysis revealed that there was a mix of components in the crude extracts, such as ellagic acid, palmitic acid, oleic acid, stearic acid, and 9(10)-EpODE. Furthermore, it could be observed that ellagic acid extracted from gallnuts contained toxic substances such as anacardic acid and ginkgolic acid (15:1). These components could be used to effectively distinguish the origin of ellagic acid from pomegranate peels or gallnuts. Additionally, a rapid quantitative analysis method using UHPLC-ESI-MS with multiple reaction monitoring (MRM) mode was developed for the quality control of ellagic acid products, by quantifying anacardic acid and ginkgolic acid (15:1). It was found that one of three ellagic acid health care products contained ginkgolic acid (C15:1) and anacardic acid at more than 1 ppm.

## 1. Introduction

Ellagic acid is a polyphenolic compound with strong tanning properties. It can combine with various biomacromolecules such as proteins and collagen to form stable tanning complexes, thereby exerting its effects [1,2,3]. Ellagic acid has astringent properties on skin tissue, which can tighten pores, reduce sebum secretion, and improve skin greasiness [4]. In addition, ellagic acid exhibits various biological activities such as antibacterial [5], antioxidant [6], and anti-inflammatory effects [7,8,9]. These advantages have led to widespread applications of ellagic acid in the fields of medicine, cosmetics, and food. For example, in the cosmetics industry, ellagic acid is widely used in skincare products, cosmetics, and oral care products [10]. In the field of medicine, researchers have found that ellagic acid has various effects such as anti-tumor [11], anti-diabetic [12], and anti-cardiovascular disease [13] activities, showing potential for disease treatment and prevention. In addition, ellagic acid has been found to enhance neuronal vitality and reduce neuronal defects in neurodegenerative diseases such as Alzheimer’s disease, Parkinson’s disease, and cerebral ischemia, demonstrating its neuroprotective effects [14,15]. Significant progress has been made in the research of ellagic acid, and its extraction, preparation, and health benefits are receiving increasing attention.

Currently, the preparation methods of ellagic acid mainly include direct extraction, natural product degradation, and chemical synthesis or chemical degradation [16,17,18,19]. Chemical synthesis or degradation require high technical expertise and equipment support. Currently, researchers are striving to find efficient, environmentally friendly, and cost-effective preparation methods to meet market demands. Natural extraction involves tannic acid hydrolysis and ellagic acid extraction from plants or animals, such as pomegranate peels (*Punica granatum* L.) [17] and gallnuts (*Rhus chinensis* Mill.) [18]. A simple one-step purification using liquid–liquid extraction with the 10% *v*/*v* water in methanol can be used for preparing pomegranate peel extract rich in ellagic acid [20]. Studies have shown that ellagic acid extracted from pomegranate peels can be consumed and is relatively safe and non-toxic to the human body, with topical anti-inflammatory and analgesic activities [21,22]. However, ellagic acid prepared from gallnuts, a traditional source, is limited to external use.

Due to the affordability of crude ellagic acid extracted from gallnuts, there is market confusion stemming from the practice of blending pomegranate peels with gallnut-derived ellagic acid to falsely present it as pomegranate-peel-derived ellagic acid. However, crude ellagic acid extracted from gallnuts contains certain toxic substances, posing safety risks. Therefore, it is crucial to conduct the strict sampling and testing of ellagic acid products to ensure the authenticity and reliability of their sources and ingredients. Additionally, conducting analyses on the toxic components of crude ellagic acid extracted from gallnuts is essential to ensure the safety of ellagic acid products. However, there are currently no effective methods to confirm the source of ellagic acid, and there is limited understanding of the components of ellagic acid prepared from pomegranate peels and gallnuts.

Therefore, this study establishes an ultra-high-performance liquid chromatography–electrospray ionization–high-resolution mass spectrometry (UHPLC-ESI-HR-MS) method to identify the components of crude ellagic acid extracts from pomegranate peels and gallnuts, as well as the differential components for distinguishing the source of ellagic acid. Based on the data, we further developed a rapid quantitative analysis method using UHPLC-ESI-MS with multiple reaction monitoring (MRM) mode for the quality control of ellagic acid products.

## 2. Results and Discussions

### 2.1. Qualitative Analysis of Crude Extracts from Pomegranate Peels and Gallnuts by UPLC-ESI-HR-MS/MS

**Figure 1:**
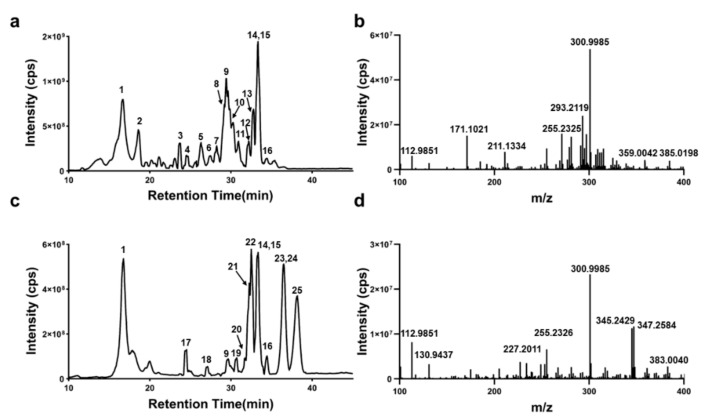
Total ion chromatograms (**a**,**c**) and the corresponding mass spectra (**b**,**d**) of ellagic acid extracted from pomegranate peel (**a**,**b**) or gallnut (**c**,**d**) in negative ion detection mode.

First, chromatographic and mass spectrometric conditions were optimized for the analysis of ellagic acid crude extracts from pomegranate peels and gallnuts. The optimized chromatographic conditions achieved baseline separation of the components, and the components showed good mass spectrometric signal responses in negative ion detection mode. The representative total ion chromatograms (Figure 1a,b) and corresponding mass spectra (Figure 1c,d) of the ellagic acid crude extracts from pomegranate peels and gallnuts are shown in Figure 1. The blank chromatogram, as Supplementary Material (Figure S1), was also provided, showing that no other matrix components eluded at the same time as the analyte. Based on the molecular ion information of the components and the fragment ion information provided by the MS/MS spectra, qualitative analysis of the sample components was performed by combining the literature and the ChemSpider database.

**Figure 2:**
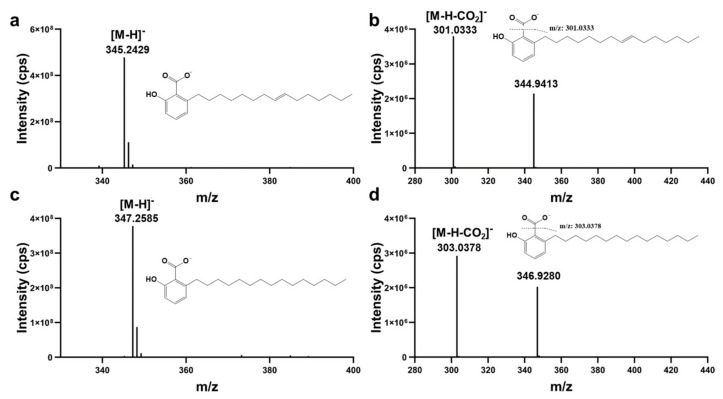
Mass spectra (**a**,**c**) and MS/MS spectra of components with RT (retention time, min) at 36.49 (**a**,**b**) and at 38.18 (**c**,**d**) in negative ion detection mode. cps: counts per second.

Two compounds (peak 23 (Rt, 36.49 min) and peak 25 (Rt, 38.18 min)) were selected from the crude extracts of ellagic acid from gallnuts as examples to illustrate structural elucidation. Figure 2 shows the MS and MS/MS spectra corresponding to compounds **23** and **25**. The *m*/*z* values for compounds **23** and **25** were 345.2429 and 347.2584, respectively. Based on high-resolution data using Xcalibur 3.0 software, the most probable molecular formula for compound **23** was calculated to be C_22_H_33_O_3_ (with a mass error of 1.27 ppm), and for compound **25** it was C_22_H_35_O_3_ (with a mass error of 1.09 ppm). In the MS/MS spectra, the neutral loss of CO_2_ from the molecular ions of both compounds ([M−H−CO_2_]^-^) resulted in fragment ions with *m/z* values of 300.9903 and 346.9168, suggesting the possible presence of carboxylic acid groups in the structures of the two compounds. It has been reported in the literature that *Rhus succedanea*, a member of the Anacardiaceae family, is the host plant of gallnuts and contained phenolic acid compounds [23,24]. Based on this information, a search in ChemSpider yielded 507 possible structures, and further screening based on the presence of carboxylic acid functional groups narrowed down the potential compounds to 20. Among these candidate structures, ginkgolic acid (15:1) and anacardic acid, which were consistent with the reported phenolic acid structures in the host plants of gallnuts, were identified.

We further purchased standard samples for UPLC-ESI-HR-MS/MS analysis, and found that their retention times and both the MS and MS/MS spectra were matched with those of compounds **23** and **25**. Therefore, it was confirmed that compound **23** was ginkgolic acid (C15:1) and compound **25** was anacardic acid.

Through the above strategies, the main components of crude extracts of pomegranate peels and gallnuts were identified, as shown in Table 1 and Table 2. Based on the data, there were a total of five mutual components in the crude extracts, namely, ellagic acid, palmitic acid, oleic acid, stearic acid, and 9(10)-EpODE, with certain variations in their quantities. Furthermore, it could be observed that both of the crude extracts contained specific components, such as ginkgoic acid, anacardic acid, and ginkgol from gallnuts. Based on these findings, we further conducted research to differentiate the source of ellagic acid from either pomegranate peels or gallnuts.

### 2.2. Identification of a Biomarker for the Discrimination of Ellagic Acid Extracted from Pomegranate Peel or Gallnuts

**Figure 3:**
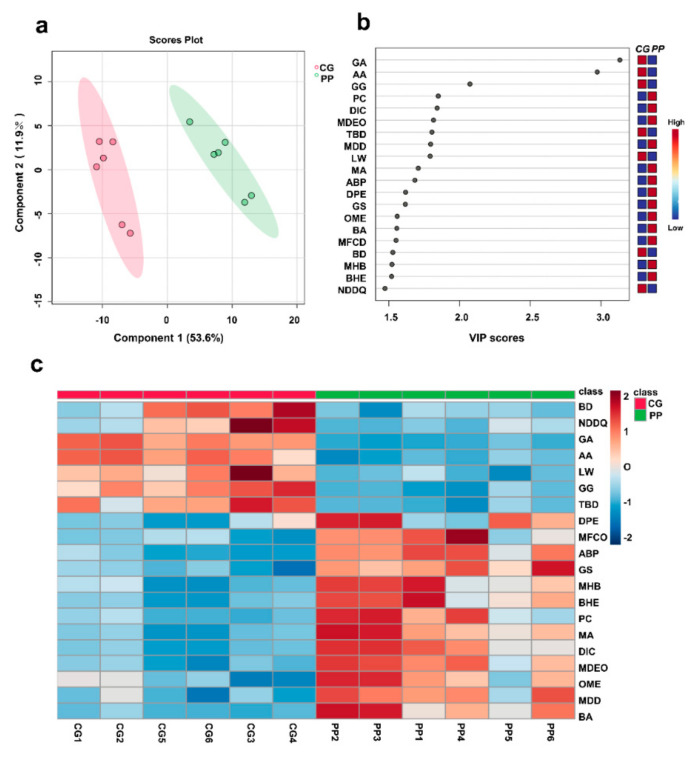
Scores plot (**a**) and VIP scores (**b**) of PLS-DA for ellagic acid extracted from gallnut and pomegranate peel, and (**c**) a heatmap of the top 20 compounds with VIP ≥1.5. Abbreviations: CG, gallnut; PP, pomegranate peel; GA, ginkgoic acid; AA, anacardic acid; GG, ginkgol; PC, 2-pentadecanone; DIC, 3,5-dimethyl-N′-[4-(trifluoromethyl) benzoyl]isoxazole-4-carbohydrazide; MDEO, Methyl 7,12-diacetoxy-3-ethoxycholan-24-oate; TBD, 5,5′,7,7′-tetrahydroxy-2,2′-bis(4-hydroxyphenyl)-4H,4′H-[8,8′-bichromene]-4,4′-dione; LW, LW8000000; MDD, 8-(4-Ethyl-1-piperazinyl)-3-methyl-7-(3-methylbutyl)-3,7-dihydro-1H-purine-2,6-dione; MA, medicagenic acid; ABP, 1-(1-Azepanyl)-3-{(3S,4R)-1-benzyl-4-[4-(2-methoxyphenyl)-1-piperazinyl]-3-piperidinyl}-1-propanone; DPE, 2,3-dinor Prostaglandin E1; GS, Glycol stearate; OME, 5-O-methyl embelin; BA, Butyldiglycol acetate; MFCD, MFCD00083370; BD, 2,1,3-Benzoxadiazole-4,7-dicarbonitrile; MHB, 4-Methoxyphenyl 4-((6-hydroxyhexyl) oxy)benzoate; BHE, (3beta,9xi)-3-(beta-D-Glucopyranosyloxy)-14-hydroxycard-20(22)-enolide; NDDQ, 7-Nitro-2,3-dioxo-2,3-dihydro-6-quinoxalinecarbonitrile.

The mass spectrometry data of crude extracts of pomegranate peel (6 samples) and gallnuts (6 samples) were imported into MetaboAnalyst 6.0 software (https://www.metaboanalyst.ca/, Access date: 25 October 2023.) for partial least squares discriminant analysis (PLS-DA). The analysis revealed distinct differences in the composition of ellagic acid prepared from pomegranate peels and gallnuts (Figure 3a). Based on the VIP scores, the top 20 compounds with VIP ≥1.5 (Figure 3b) were selected and heatmap analysis was performed (Figure 3c). It was found that anacardic acid (AA), ginkgolic acid (C15:1) (GA), and ginkgol (GG) were specific compounds for ellagic acid prepared from gallnuts. Among them, anacardic acid and ginkgolic acid (C15:1) showed the largest differences.

The recently discovered ginkgolic acid could be mainly divided into five types: ginkgolic acid (C13:0), ginkgolic acid (C15:1), ginkgolic acid (C17:2), ginkgolic acid (C15:0) (anacardic acid), and ginkgolic acid (C17:1). Ginkgolic acid has potential sensitizing and mutagenic effects, as well as strong cytotoxicity [25,26,27]. It can cause severe allergic reactions, genetic mutations, and neurotoxicity, leading to nausea, gastric burning sensation, anaphylactic shock, allergic purpura, exfoliative dermatitis, allergic reactions in the gastrointestinal mucosa, spasms, and neurological paralysis. Due to the adverse effects of ginkgolic acid, many pharmacopoeias have set limits on the content of ginkgolic acid in extracts, with a maximum allowable limit of 5 mg/kg [28,29]. Therefore, the rapid detection of anacardic acid and ginkgolic acid (C15:1), the differential components of ellagic acid prepared from gallnuts, was particularly important. This study continued to develop a quantitative analysis method for ginkgolic acid.

### 2.3. Quantitative Analysis of Anacardic Acid and Ginkgolic Acid (C15:1)

**Figure 4:**
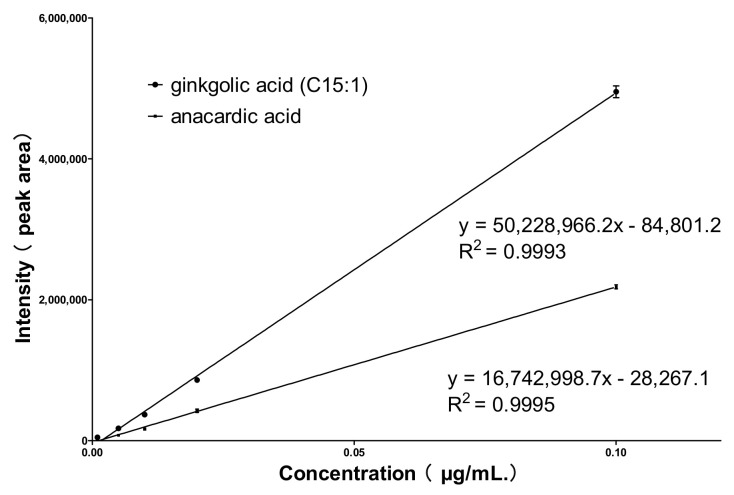
Standard curves of ginkgolic acid (C15:1) and anacardic acid.

Qtrap 4500 mass spectrometry in negative ion detection mode, with a high-sensitivity multiple reaction monitoring (MRM) mode for data acquisition, was performed. Firstly, the mass spectrometry conditions were optimized using standard solutions, with 345.2→301.2 and 347.2→303.2 as the ion pairs for the quantification of anacardic acid and ginkgolic acid (C15:1), respectively. Analysis was performed according to the optimized conditions, and calibration curves for both compounds were constructed, as shown in Figure 4. The R^2^ values were all greater than 0.99, indicating good linearity between the signal intensity and the concentration. The mixed control solution was diluted in a gradient of mass concentrations from high to low, and quantities of each control sample at signal-to-noise ratios of S/N = 10 and S/N = 3 were taken as the quantitation limit and detection limit, respectively. The limit of detection (LOD) was found to be 0.001 μg/mL, and the limit of quantification (LOQ) was 0.005 μg/mL. When detecting 0.02 μg/mL ginkgolic acid (C15:1) and anacardic acid, this method was highly accurate and reliable with <10.0% error, <7.2% relative intra-day standard deviation, and <3.7% relative inter-day standard deviation. The complete validation data are shown in Table S1.

According to the above method, samples of gallnut-prepared ellagic acid (six samples) as well as three ellagic acid health care products purchased from the market were analyzed, as shown in Table 3. The analysis data showed that the contents of ginkgolic acid (C15:1) and anacardic acid in gallnut-prepared ellagic acid from different manufacturers and batches varied greatly. More importantly, among the three ellagic acid health care products purchased from the market, one of them contained ginkgolic acid (C15:1) and anacardic acid in excess of 1 ppm, which was substandard, although it did not exceed the rules. Therefore, it is very important to conduct strict sampling and testing of ellagic acid products to ensure the authenticity and reliability of their sources and ingredients.

## 3. Materials and Methods

### 3.1. Materials and Reagents

Methanol (MeOH, HPLC grade) and acetonitrile (ACN, HPLC grade) were purchased from Thermo Fisher Scientific (Waltham, MA, USA). The anacardic acid (purity: 99%, cas: 16611-84-0) and ginkgolic acid (15:1) (purity: 99%, cas: 22910-60-7) standards were purchased from Beijing Bailinwei Technology Co., Ltd. The six pomegranate peel ellagic extracts (sample codes: PP-1, PP-2, PP-3, PP-4, PP-5, PP-6; picking date: September–October, 2022) and six gallnut ellagic extracts (sample codes: CG-1, CG-2, CG-3, CG-4, CG-5, and CG-6; picking date: June–October, 2022) were provided by four companies (Anhui or Shanxi or Hebei, China); three ellagic acid health care products (sample codes: HCP-1, HCP-2, and HCP-3) were purchased from the market (Table 4 for detailed information). The water was purified using a Milli-Q system from Merk (Kenilworth, NJ, USA).

### 3.2. Solution Preparation of Crude Extracts and Health Care Products

The extraction of ellagic acid was performed in accordance with the previously published method, with some modification [20]. In detail, 1 g samples of ellagic acid crude extracts or health care products were accurately weighed into a centrifuge tube (50 mL). Then, 10 mL of MeOH was added and vortex-mixed for 1 min. Ultrasonic extraction performed for 30 min (45 °C, 40 KHz). Centrifugation at 10,000× *g* was conducted for 10 min to collect the supernatant. Finally, the supernatant was filtered through a 0.22 μm membrane before UHPLC-ESI-HR-MS analysis.

### 3.3. Preparation of Standard Solution

The ginkgolic acid (15:1) and anacardic acid standard substances were dissolved in MeOH to prepare a concentrated mixed standard solution with a concentration of 5 μg/mL. Then, the solution was sequentially diluted with MeOH to prepare solutions with concentrations of 1 μg/mL, 0.5 μg/mL, 0.2 μg/mL, 0.1 μg/mL, and 0.02 μg/mL for later use.

### 3.4. Ultra-High-Performance Liquid Chromatography–Electrospray Ionization–High-Resolution Mass Spectrometry (UHPLC-ESI-HR-MS)

To qualitatively analyze ellagic acid extracts from pomegranate peels and gallnuts, samples were loaded using a Thermo U3000 LC system (Thermo Fisher Scientific, USA) equipped with an auto sampler. A BEH C18 reversed-phase column (1.7 μm, 2.1 mm ID × 100 mm, Waters, Milford, MA, USA) was utilized, then maintained at 35 °C with the sample tray set at 10 °C. Mobile phases A and B were H_2_O and ACN, respectively, and the UHPLC separations were conducted over 62 min/sample using the following gradient: 0–3 min, 3% B; 3–30 min, 3–97% B; 30–40 min, 97% B; 40–60 min, 97–5% B; 60–62 min, 3% B. All transitions were linear, and the flow rate was set at 200 μL/min, with 1 μL samples injected for analysis. The UHPLC system was coupled to an Orbitrap Fusion Lumos mass spectrometer (Thermo Fisher Scientific, USA) with an electrospray ionization (ESI) source for the MS and MS/MS analyses. The mass spectrometry system operated in negative ion detection mode, with the simultaneous acquisition of MS and MS/MS data. The scanning range of was set from 100 to 1500 *m*/*z*, the temperature of the ion transfer tube was 350 °C, the temperature of the atomizer was set at 300 °C, the spray voltage was −3500 V, and the high-energy collision dissociation method (HCD) with a collision energy of 35 ± 15% was used for obtaining MS/MS data.

For the quantitative analysis of anacardic acid and ginkgolic acid (15:1), samples were loaded through an LC system (I-class Acquity UHPLC, Waters) equipped with an auto sampler. The UHPLC system was connected to a 4500 QTRAP mass spectrometer (SCIEX) with an ESI source for the MS analysis. Both the nebulizer and desolvation gases used were nitrogen. Typical operating parameters were set as follows: curtain gas (CUR) 25; collision gas (CAD) medium; temperature, 550 °C; ion source gas 1 (GS1) 45; ion source gas 2 (GS2) 50; and electrospray voltage, −4500 V. For the qualification, an instrumental method in multiple reaction monitoring (MRM) mode was established. The anacardic acid and ginkgolic acid (15:1) parent ions in Q1 (347.2 and 345.2, respectively) and their characteristic fragment ions in Q3 (303.2 and 301.2, respectively) were used as ion pairs in MRM survey channels. The collision energy was set as −35 V. The peak areas of anacardic acid and ginkgolic acid (15:1) obtained by QTRAP 4500 were collected for calculating the concentrations according to the standard curve.

### 3.5. Data Processing for the Identification of Ellagic Acid Extracts from Pomegranate Peels or Gallnuts

Data obtained by Orbitrap Fusion Lumos MS were processed using Xcalibur and Mass Frontier 7.0 (Thermo Fisher Scientific, USA). The compounds were identified by online searching (Chemspider, http://www.chemspider.com/Default.aspx, Access date: 20 March 2023), to precisely match the accurate molecular ion obtained by MS (mass error < 5 ppm), combined with the fragment ions in MS/MS data obtained by high-energy collisional dissociation (HCD). Moreover, standards were also used for the confirmation of structures.

To identify biomarkers for the discrimination of ellagic acid extracted from pomegranate peels or gallnuts, as described in detail previously [30], orthogonal partial least square discriminant analysis (OPLS-DA) was used; the discriminating variables were selected according to variable importance in projection (VIP) >1.5.

## 4. Conclusions

Previous studies have often focused on the extraction and efficacy validation of active components from pomegranate peels or gallnuts. Our work emphasizes the analysis of the differences in the components of the extracts from both sources, and the ability to distinguish from which source the ellagic acid extracts originated. We combined non-targeted and targeted UHPLC-ESI-MS analysis techniques in our study, resulting in highly reliable results. The experiment has established a method based on UHPLC-ESI-HR-MS combined with multivariate statistical analysis techniques to analyze the differences in chemical composition between gallnut-extracted ellagic acid and pomegranate-peel-extracted ellagic acid; it was observed that ellagic acid extracted from gallnuts contained toxic substances such as anacardic acid and ginkgolic acid (15:1), which is a new finding. Furthermore, we confirmed the presence of these two compounds using UHPLC-ESI-MS and standard reference comparison. The identified ginkgolic acid (C15:1) and anacardic acid could be used to effectively distinguish the origin of ellagic acid from either pomegranate peels or gallnuts. Furthermore, a multiple reaction monitoring (MRM) method was established for the rapid quantitative analysis of ginkgolic acid (C15:1) and anacardic acid in raw materials and health care products. This study presents a rapid and reliable means to differentiate between ellagic acid derived from gallnuts or pomegranate peels, offering a crucial reference to prevent the adulteration of ellagic acid and ensure the authenticity and quality of products in various industries, including food, pharmaceuticals, and cosmetics.

## Figures and Tables

**Table 1:** The 16 compounds identified from pomegranate peel extracts.

No. *	Name	RT **/min	Formula	Fragment Ions	[M−H]^-^ *m/z* Detected	[M−H]^-^ *m/z* Theoretical	Mass Error /ppm
1	Ellagic Acid	16.66	C_14_H_6_O_8_	256.9	300.9981	300.9979	0.8
2	Ethyl 4-hydroxycyclohexane carboxylate	18.64	C_9_H_16_O_3_	127.1	171.1021	171.1016	2.8
3	Traumatin	23.67	C_12_H_20_O_3_	166.9	211.1334	211.1329	2.3
4	Phloionolic Acid	24.63	C_18_H_36_O_5_	286.9	331.2483	331.2479	1.2
5	13(S)-HpOTrE	26.31	C_18_H_30_O_4_	208.8	309.2063	309.2060	1.2
6	VS1150000	28.10	C_18_H_34_O_4_	294.9	313.2374	313.2373	0.3
7	12-oxo Phytodienoic Acid	29.00	C_18_H_28_O_3_	246.9	291.1961	291.1955	2.1
8	9,10-Dihydroxystearic Acid	29.30	C_18_H_36_O_4_	297.0	315.2533	315.2530	1.1
9	9(10)-EpODE	29.49	C_18_H_30_O_3_	248.9	293.2117	293.2111	1.9
10	Ricinelaidic Acid	30.19	C_18_H_34_O_3_	279.0	297.2423	297.2424	−0.5
11	Linolenelaidic Acid	32.09	C_18_H_30_O_2_	232.9	277.2166	277.2162	1.6
12	Palmitelaidic Acid	32.39	C_16_H_30_O_2_	208.9	253.2166	253.2162	1.7
13	Linoelaidic Acid	32.74	C_18_H_32_O_2_	234.9	279.2323	279.2319	1.6
14	Palmitic Acid	33.19	C_16_H_32_O_2_	226.8	255.2324	255.2319	1.9
15	Oleic Acid	33.44	C_18_H_34_O_2_	236.9	281.2478	281.2475	1.2
16	Stearic Acid	35.28	C_18_H_36_O_2_	239.0	283.2638	283.2632	2.2

* the numbers of the peaks according to Figure 1; ** retention time.

**Table 2:** The 14 compounds identified from gallnut extracts.

No. *	Name	RT **/min	Formula	Fragment Ions	[M−H]^-^ *m/z* Detected	[M−H]^-^ *m/z* Theoretical	Error /ppm
1	Ellagic Acid	16.7	C_14_H_6_O_8_	256.9	300.9977	300.9979	−0.5
17	(−)-pinellic Acid	24.5	C_18_H_34_O_5_	228.9	329.2327	329.2323	1.1
18	1-nonanoic Acid	27.0	C_9_H_18_O_2_	128.9	157.1227	157.1223	2.4
9	9(10)-EpODE	29.6	C_18_H_30_O_3_	248.9	293.2116	293.2111	1.7
19	Lauric Acid	30.6	C_12_H_24_O_2_	155.1	199.1696	199.1693	1.9
20	PA3500000	31.8	C_23_H_32_O_2_	163.9	339.2321	339.2319	0.5
21	Myristic Acid	32.2	C_14_H_28_O_2_	183.2	227.2010	227.2006	1.7
22	Betulonic Acid	32.6	C_30_H_46_O_3_	409.1	453.3364	453.3363	0.2
14	Palmitic Acid	33.3	C_16_H_32_O_2_	226.8	255.2325	255.2319	2.5
15	Oleic Acid	33.4	C_18_H_34_O_2_	236.9	281.2478	281.2475	1.1
16	Stearic Acid	34.4	C_18_H_36_O_2_	239.0	283.2637	283.2632	1.8
23	Ginkgoic Acid	36.5	C_22_H_34_O_3_	301.0	345.2428	345.2424	1.1
24	Ginkgol	37.1	C_21_H_34_O	283.1	301.2539	301.2526	4.4
25	Anacardic Acid	38.2	C_22_H_36_O_3_	303.0	347.2585	347.2581	1.1

* the numbers of the peaks according to Figure 1; ** retention time.

**Table 3:** The content of ginkgolic acid (C15:1) and anacardic acid from different companies.

Sample	Ginkgolic Acid (mg/kg *) Mean ± SD **	Anacardic Acid (mg/kg *) Mean ± SD **
CG-1	4.46 ± 0.06	9.26 ± 0.01
CG-2	4.27 ± 0.37	8.96 ± 0.49
CG-3	3.02 ± 0.10	8.78 ± 0.22
CG-4	0.84 ± 0.05	2.61 ± 0.10
CG-5	0.93 ± 0.01	3.15 ± 0.04
CG-6	4.30 ± 0.06	9.12 ± 0.04
HCD-1	1.43 ± 0.09	1.69 ± 0.08
HCD-2	0.06 ± 0.03	0.11 ± 0.05
HCD-3	0	0

* units per mass of product in dry basis; ** 3 repeated measurements.

**Table 4:** Information on the 3 health care products.

Sample/Place of Production	Date of Manufacture	Origin	Nature	Quantity of Ellagic Acid Informed	Other Ingredients
HCD-1 America	March 2020	Pomegranate seed extract	Dry power in capsule	70%	Silica, vegetable magnesium stearate, vegetable capsule
HCD-2 America	July 2022	Pomegranate fruit extract	Dry power in capsule	40%	Cellulose, vegetable Cellulose Capsule
HCD-3 Germany	April 2022	Pomegranate fruit extract	Dry power in capsule	40%	microcrystalline cellulose, gelatin

## Data Availability

The data presented in this study are available in article and Supplementary Materials.

## Supplementary Materials

The following supporting information can be downloaded at: https://www.mdpi.com/article/10.3390/molecules29030666/s1, Figure S1. The total ion chromatograms of blank. Table S1. The result when detecting 0.02 μg/mL ginkgolic acid (C15:1) and anacardic acid.
