# Supplementary material for: Ultra-High-Performance Liquid Chromatography–Electrospray Ionization–High-Resolution Mass Spectrometry for Distinguishing the Origin of Ellagic Acid Extracts: Pomegranate Peels or Gallnuts
Source: Molecules. 2024 Jan 31;29(3):666. doi: 10.3390/molecules29030666 (PMC10856690; doi:10.3390/molecules29030666)
Supplement: Supplementary file 1 [file molecules-29-00666-s001.zip › molecules-2777826-supplementary.pdf]

Supplementary materials

# Ultra-High-Performance Liquid Chromatography–Electrospray Ionization–High-Resolution Mass Spectrometry for Distinguishing the Origin of Ellagic Acid Extracts: Pomegranate Peels or Gallnuts

**Jinchao Wei [1,2], Renjian Xu [3], Yuanyuan Zhang [1,2], Lingyu Zhao [1,2], Shumu Li [1] and Zhenwen Zhao [1,2,*]**

[1] Beijing National Laboratory for Molecular Sciences, CAS Research/Education Center for Excellence in Molecular Sciences, Key Laboratory of Analytical Chemistry for Living Biosystems, Beijing Mass Spectrum Center, Institute of Chemistry, Chinese Academy of Sciences, Beijing 100190, China; weijinchao@iccas.ac.cn (J.W.); zhangyuanyuan@iccas.ac.cn (Y.Z.); zhaolingyu@iccas.ac.cn (L.Z.); lishumu@iccas.ac.cn (S.L.)

[2] Graduate School, University of Chinese Academy of Sciences, Beijing 100049, China

[3] Anhui Deren Biotechnology Co., Ltd., Suzhou 234122, China; xrj.hbzy@163.com

* Correspondence: zhenwenzhao@iccas.ac.cn

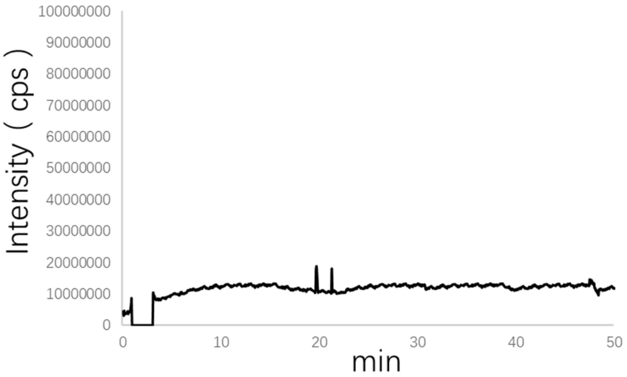


**Figure S1.** The total ion chromatograms of blank.

**Table S1.** The result when detecting 0.02 μg/mL ginkgolic acid (C15:1) and anacardic acid.

| | Ginkgolic acid (C15:1) | | | | Anacardic acid | | | |
|---|---|---|---|---|---|---|---|---|
| | Intra day | | | Inter day | Intra day | | | Inter day |
| | Day 1 | Day 2 | Day 3 | | Day 1 | Day 2 | Day 3 | |
| Mean | 0.019 | 0.018 | 0.018 | 0.019 | 0.021 | 0.022 | 0.022 | 0.022 |
| RSD | 3.9 | 2.4 | 2.6 | 3.7 | 7.2 | 5.6 | 3.8 | 2.3 |
| Error | -3.2 | -8.1 | -9.0 | 6.8 | 5.9 | 9.8 | 10.0 | 8.5 |
